# Multiple Autoimmune Syndromes Including Acute Disseminated Encephalomyelitis, Myasthenia Gravis, and Thyroiditis Following Messenger Ribonucleic Acid-Based COVID-19 Vaccination: A Case Report

**DOI:** 10.3389/fneur.2022.913515

**Published:** 2022-05-27

**Authors:** Khouloud Poli, Sven Poli, Ulf Ziemann

**Affiliations:** ^1^Department of Neurology and Stroke, Eberhard-Karls University, Tübingen, Germany; ^2^Hertie Institute for Clinical Brain Research, Eberhard-Karls University, Tübingen, Germany

**Keywords:** multiple autoimmune syndrome, ADEM, thyroiditis, myasthenia gravis, mRNA-based COVID-19 vaccines

## Abstract

The global pandemic has resulted from the emergence of severe acute respiratory syndrome coronavirus-2 (SARS-CoV-2), causing coronavirus disease 2019 (COVID-19). To control the spread of the pandemic, SARS-CoV-2 vaccines have been developed. Messenger ribonucleic acid (mRNA)-based COVID-19 vaccines have been the most widely used. We present the case of a 65-year-old patient, who was diagnosed with acute disseminated encephalomyelitis, ocular myasthenia gravis, and autoimmune thyroiditis, following his third mRNA COVID-19 vaccination. On admission, the patient showed mild left-sided hemiparesis, contralateral dissociated sensory loss, dizziness, and right-sided deafness. Brain MRI revealed multiple acute inflammatory contrast-enhancing periventricular and brainstem lesions with involvement of vestibulo-cerebellar tract and cochlear nuclei. Despite steroid pulse and intravenous immunoglobulin therapy, clinical symptoms and MRI lesions worsened, and additional signs of ocular myasthenia gravis and elevated but asymptomatic thyroid antibodies developed. After repeated plasma exchange, all clinical symptoms resolved. This is, to the best of our knowledge, the first case report of multiple autoimmune syndromes triggered by COVID-19 vaccination. The rare occurrence of such treatable autoimmune complications should not question the importance of vaccination programs during the COVID-19 pandemic.

## Introduction

Besides specific vaccine complications (such as vaccine-induced thrombotic thrombocytopenia after vector-based COVID-19 vaccines), the association between new-onset autoimmune disease and vaccination could not be established yet, most likely due to low incidence. However, cases of vaccine-triggered autoimmune phenomena have been reported, and different mechanisms have been suggested (molecular mimicry, production of autoantibodies, and vaccine adjuvants) (1). Acute disseminated encephalomyelitis (ADEM) is an autoimmune demyelinating disease that affects multiple areas of the central nervous system and typically presents with multifocal neurologic symptoms. It is commonly considered a monophasic disease with a rare recurrent or multiphasic variant (2). Up to three-quarters of ADEM events are associated with viral infections (3). Prior immunizations may also trigger ADEM events (4). A causal relationship between inactivated and mRNA SARS-CoV-2 vaccination has been reported (5–8). Myasthenia gravis due to acetylcholine receptor (AChR) autoantibodies, which prevent transmission of the excitatory cascade at the neuromuscular junction during muscle contraction, maybe rarely, also induced by mRNA COVID-19 vaccination (9). Likewise, cases of autoimmune thyroiditis have been described following exposure to inactivated and mRNA-based SARS-CoV-2 vaccines (10).

We report a patient, who developed all of these three autoimmune disorders shortly after being vaccinated for SARS-CoV-2.

## Case Description

A 65-year-old male patient was referred for the subacute onset of paresis of the left arm, followed by loss of pain and temperature sensation on the right side of the body, as well as right-sided deafness with vertigo, 3 days after receiving the third dose of the mRNA-based Pfizer-BioNTech COVID-19 vaccine, without any acute allergic reactions.

His medical history was relevant for multiphasic ADEM, with two previous clinical episodes, 10 and 11 years prior to this admission. In the first event, the patient manifested mild right-sided sensorimotor hemiparesis and Th11/Th12 paraplegia with urinary incontinence in the second event. At that time, cerebral and spinal cord magnetic resonance imaging (MRI) showed multiple T2-weighted hyperintense lesions involving supratentorial areas and, respectively, the spinal cord, all with T1 contrast enhancement. Cerebrospinal fluid (CSF) showed lymphocytic pleocytosis (50 and 7 cells/mm^3^, respectively), while protein and glucose levels were within reference ranges. Oligoclonal banding was not present in both events. Confirming the diagnosis of ADEM, stereotactic brain biopsy showed typical perivenous inflammatory demyelination. The patient fully recovered under intravenous high-dose corticosteroids both times. A Follow-up MRI of the brain solely showed minor periventricular white matter sequelae; spinal cord lesions were completely resolved.

The patient's family history was positive for Graves' disease by a daughter; further (auto)immune disorders or neurological diseases were denied.

On the current admission, the patient was alert and oriented and presented mild left-sided hemiparesis (MRC 4/5) with contralateral dissociated sensory loss, and right-sided vestibulocochlear nerve deficit. Brain MRI revealed acute inflammatory gadolinium-enhancing lesions on the right cerebellar peduncle, as well as pons and medulla oblongata. CSF analysis showed lymphocytic pleocytosis (54 cells/mm^3^), while protein and glucose levels were normal. Oligoclonal bands were searched in serum and CSF by isoelectric focusing, with negative results (type 1 pattern). Screening for bacterial, viral, and fungal neuro infections was negative. Tests were also negative for antibodies targeting antigens associated with demyelinating disorders of the central nervous system (myelin oligodendrocyte protein and aquaporin-4), as well as onconeural-, and anti-ganglioside antibodies. The CSF cytological analysis excluded circulating malignant cells. Biochemical serum markers for sarcoidosis (angiotensin-converting enzyme and soluble interleukin-2 receptor) were unremarkable, and CD4/CD8 ratio in CSF and bronchoalveolar lavage were not elevated. Interleukin-10 in CSF was normal, and chemokine CXC ligand 13 slightly increased. Complete blood count and markers of systemic autoimmunity (including antinuclear, extractable nuclear antigen, anti-neutrophil cytoplasmic, and antiphospholipid antibodies, as well as complement C3 and C4) were normal/negative.

**Figure 1:**
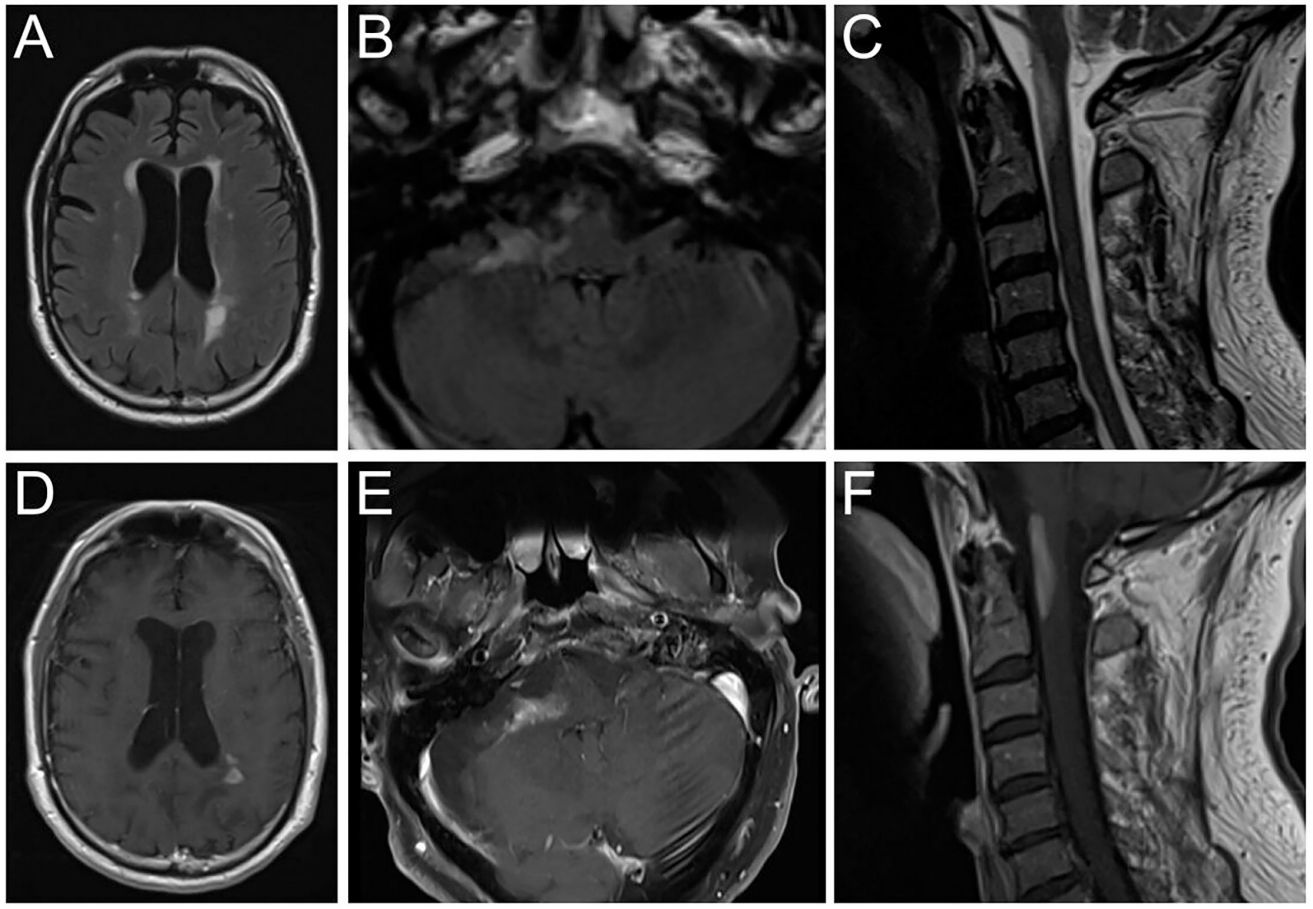
Brain magnetic resonance imaging showing FLAIR hyperintense lesions **(A–C)** and T1 contrast-enhancement **(D–F)** in the periventricular white matter **(A,D)**, right cerebellar peduncle **(B,E)** and medulla oblongata/upper cervical spinal cord **(C,F)**. FLAIR, Fluid attenuated inversion recovery.

The patient was treated with high-dose intravenous methylprednisolone (1 g daily) for five days. Due to non-response, intravenous immunoglobulin therapy with a total dose of 2 g/kg, fractionated in 5 days, was started. Rapid clinical deterioration with the development of severe left-sided hemiparesis (MRC 2/5), hemiataxia, and major difficulties to walk was accompanied by new periventricular and progressive infratentorial and upper cervical spinal cord contrast-enhancing lesions on follow-up MRI (Figure 1).

Furthermore, the patient developed fluctuating binocular horizontal diplopia and ptosis of the right eye, with worsened toward the end of the day. Diagnostic pyridostigmine (60 mg orally) did not improve ocular symptoms within a 1-h observation period. Immunologic testing showed elevated anti-AChR antibody titers (2.1 nmol/L, normal range is <0.4). Autoantibodies against muscle-specific kinase and titin were negative. No thymoma was detected on chest computed tomography (CT). The patient was newly diagnosed with ocular myasthenia gravis and started on oral pyridostigmine 90 mg twice a day.

On further laboratory investigations, positive anti-thyroglobulin antibodies (21.4 IU/ml, normal range is <4.5), anti-thyroid peroxidase antibodies (197.9 KU/L, normal range is <60), and anti-thyroid stimulating hormone (TSH) receptor autoantibodies (2.09 IU/L, normal range is 1.75) were detected. Thyroid function (TSH, triiodothyronine, and thyroxine), however, was normal, with no past medical history of thyroid disease. Thyroid ultrasonography was normal. Subacute thyroiditis was first diagnosed based on the patient's laboratory findings.

Considering the clinical worsening and the development of multiple autoimmune disorders despite treatment with corticosteroids and intravenous immunoglobulins, plasmapheresis was indicated. Seven plasma exchanges were conducted within 13 days. The hemiparesis improved, and the patient regained walking ability. Follow-up MRI brain and spinal cord scans after the third plasma exchange already revealed reduced lesion size and contrast enhancement. Ocular myasthenic symptoms resolved completely. Anti-thyroid and anti-AChR autoantibodies were no longer detectable. The patient was referred to rehabilitation, where clinical status further improved until 1 month after plasma exchange (three months after onset).

## Discussion

Our patient met the diagnostic criteria for ADEM set by the International Pediatric MS Study Group (2), and alternative diagnoses, such as infectious or another autoimmune encephalitis, were excluded. ADEM, following vaccination, is a well-known entity and has been reported after mRNA SARS-CoV-2 vaccination (Moderna and BioNTech/Pfizer), even among older adults such as our patient (5–7), but also after inactivated vaccine (Sinovac) (8). Previously reported cases of vaccination-triggered ADEM had an excellent response to systemic corticosteroids and/or intravenous immunoglobulins. Our patient, however, deteriorated under first-line therapy and required plasmapheresis. Steroid resistance is commonly observed in cases like ours with fulminant and/or multiphasic ADEM (11, 12). Moreover, our patient simultaneously developed two other autoimmune disorders, i.e., ocular myasthenia gravis and subacute thyroiditis. In contrast to ADEM, these were first-in-life episodes. Both have been separately described following both mRNA (BioNTech/Pfizer) (9, 10) and inactivated (Sinovac) (13) SARS-CoV-2 vaccination. A literature search, however, did not identify any case with a multiple autoimmune syndromes similar to ours. The missed opportunity of testing neutralizing antibodies against SARS-CoV-2 in serum and/or CSF before immunoglobulin therapy and plasmapheresis may be considered a limitation of our case study. The presence of these in either compartment, however, would not have proven the causal link with autoimmune reaction.

The rare occurrence and favorable outcomes of vaccination-triggered ADEM, myasthenia gravis, and subacute thyroiditis, as well as the fact that severe (multiple) autoimmune syndromes may also occur after COVID-19 infection (14–17), do not detract from the public health imperative to vaccinate against COVID-19. However, clinicians should be aware that those autoimmune diseases can potentially occur alone or simultaneously, following both mRNA-based and inactivated SARS-CoV-2 vaccines, and may affect patients of any age. Extended half-life monoclonal neutralizing antibodies against SARS-CoV-2 may be considered to protect patients with insufficient immunity, in whom further vaccines are not advised (18).

## Data Availability Statement

The original contributions presented in the study are included in the article, further inquiries can be directed to the corresponding author.

## Ethics Statement

Ethical review and approval was not required for the study on human participants in accordance with the local legislation and institutional requirements. The patients/participants provided their written informed consent to participate in this study.

## Author Contributions

KP conducted the literature search and drafted the first version of the manuscript. SP and UZ made critical revisions. All authors approved the submitted version of the manuscript.

## Conflict of Interest

The authors declare that the research was conducted in the absence of any commercial or financial relationships that could be construed as a potential conflict of interest.

## Publisher's Note

All claims expressed in this article are solely those of the authors and do not necessarily represent those of their affiliated organizations, or those of the publisher, the editors and the reviewers. Any product that may be evaluated in this article, or claim that may be made by its manufacturer, is not guaranteed or endorsed by the publisher.
